# Long-term disease-specific quality of life after laparoscopic Nissen fundoplication in patients with borderline GERD

**DOI:** 10.1007/s00464-024-11176-0

**Published:** 2024-08-19

**Authors:** Theresa N. Wang, Anahita D. Jalilvand, Shuchi Sharma, Bryan W. An, Kyle A. Perry, Patrick J. Sweigert

**Affiliations:** 1 Department of Surgery, Center for Minimally Invasive Surgery, The Ohio State University, 395 W 12th Ave Suite 670, Columbus, OH 43210 USA; 2 Division of Trauma, Critical Care and Burn, Department of Surgery, The Ohio State University, Columbus, OH USA; 3 The Ohio State University College of Medicine, Columbus, OH USA

**Keywords:** Fundoplication, GERD, Quality of life, Reflux, Acid exposure time

## Abstract

**Background:**

Historically, DeMeester score over 14.7 has been used to diagnose GERD. The 2022 American Gastroenterological Association clinical guidelines define GERD based on acid exposure time (AET) instead of DeMeester score. We aim to compare outcomes after laparoscopic Nissen fundoplication (LNF) in patients based on differing GERD diagnostic criteria.

**Methods:**

Patients who underwent first-time LNF between 2009 and 2017 were identified. Demographics, objective GERD evaluation, and outcomes were maintained in an IRB-approved database. Disease-specific quality of life was assessed with a survey (GERD-HRQL) with higher values representing more symptomatic disease. Descriptive statistics, Fischer’s exact test and logistic regression were used to analyze the data, *p*-value < 0.05.

**Results:**

225 patients were stratified into two groups: borderline GERD (AET 4–6%, *n* = 25.11%) and GERD (AET ≥ 6%, *n* = 200.89%). The mean age was 50.1 ± 13.4 years and 169 (75%) were female. Baseline GERD-HRQL was lower in the borderline group (24.3 vs 30.0, *p* = 0.031). Short-term (5 weeks [IQR 4, 8]), medium-term (14 months [IQR 7.25, 31]) and long-term (6.75 years [IQR 5.5, 8]) follow-up was performed. GERD-HRQL scores did not differ between borderline and GERD patients at short-(6.0 vs 7.1, *p* = 0.630), medium-(12.0 vs 12.1, *p* = 0.818), or long-term follow-up (10.0 vs 9.0, *p* = 0.757). The absolute long-term improvement in GERD-HRQL was −12.3 (*p* = 0.022) vs. −21.3 (*p* < 0.001). At long-term follow-up there was no difference in PPI use (50% vs 47%, *p* = 0.852), satisfaction (58% vs 76%, *p* = 0.187), willingness to repeat the procedure given the benefit of hindsight (75% vs 85%, *p* = 0.386), or need for reoperation (14% vs 13%, *p* = 0.910).

**Conclusion:**

Both patients with borderline GERD and GERD achieve GERD-HRQL improvements at 7 years following laparoscopic Nissen fundoplication and demonstrate similar long-term PPI usage and satisfaction with surgical results. Borderline GERD patients have lower GERD-HRQL at baseline, and thus have smaller improvements in their QOL scores. Anti-reflux surgery should be considered for patients with a diagnosis of borderline GERD refractory to medical therapy.

## Background

Gastroesophageal reflux disease (GERD) is estimated to have a global burden of over 700 million patients, with considerable economic and quality of life impact. Current estimates suggest an increasing incidence of GERD, due in part to increased rates of obesity [1]. While lifestyle changes and medical therapies form the foundational treatment strategy for most patients, there are a subset of patients in whom surgical therapy should be considered. For decades, minimally invasive fundoplication has been offered as a safe and effective intervention to improve patient’s GERD-related quality of life by improving symptoms, healing erosive esophagitis, and allowing for the most durable therapeutic modality to remain off of anti-secretory therapies long-term [2]. Traditionally, fundoplication has been reserved for patients with positive objective pH testing demonstrating increased acid exposure in the distal esophagus. For nearly 50 years, the objective score primarily used for this surgical decision has been a composite DeMeester score over 14.7, which is calculated based on a formula of weighted deviations from mean exposures in healthy individuals versus those with GERD [3].

More recent consensus GERD guidelines have emphasized the percent acid exposure time (AET) during objective testing as a more reliable objective measure on pH testing. In 2022, the American Gastroenterological Association (AGA) published updated clinical practice guidelines defining GERD based on AET, with AET 4–6% representing borderline GERD, and AET ≥ 6% defining the presence of GERD [4]. Specifically, these guidelines recommended that patients be considered candidates for invasive (i.e., surgical or endoscopic) anti-reflux procedures only if they were categorized as having GERD. However, discussion over the utilization of DeMeester score versus AET for diagnosis of GERD persists, and the 2022 American College of Gastroenterology clinical guidelines recommended utilizing both in clinical diagnosis [5, 6]. A 2023 consensus guideline by multiple surgical societies on the treatment of GERD recommend ambulatory pH monitoring as part of the evaluation, but did not discuss the use of DeMeester score versus AET for the diagnosis of GERD [7].

Long-term follow-up of disease-specific quality of life scores after laparoscopic fundoplication for GERD, as it has been historically diagnosed, have been demonstrated in multiple studies to result in durable improvements anywhere from one to twenty years after surgery [8–13]. A recent study comparing patient-reported quality of life scores after laparoscopic fundoplication for historical criteria versus AET-based criteria for GERD concluded that AET may better define patients who should be offered anti-reflux surgery than DeMeester criteria [14]. There remains a paucity of data comparing surgical outcomes of laparoscopic Nissen fundoplication (LNF) in patients with borderline GERD to those with GERD, particularly for extended follow-up intervals. As such, patients who have borderline GERD as defined by AET but would meet the criteria for GERD as defined by DeMeester score can be challenging to counsel given these recent guidelines. Long-term patient satisfaction and symptom improvement data is critical for patient counseling and decision-making regarding candidacy for fundoplication. This study seeks to improve understanding of the long-term patient quality of life outcomes after fundoplication in the setting of borderline AET. We hypothesized that patients with borderline GERD, when compared to those with GERD as defined by AET, would experience similar long-term improvements in disease-specific quality of life after laparoscopic Nissen fundoplication.

## Methods

### Study design and participants

We performed a retrospective cohort study of patients undergoing elective laparoscopic Nissen fundoplication by a single high-volume surgeon for reflux at the Ohio State University Wexner Medical Center between 2009 and 2017. Patients who had prior anti-reflux surgery, no pre-operative ambulatory pH testing, or pre-operative AET < 4% were excluded. Patients who were found to have paraesophageal hernia prior to or at the time of operation were also excluded. Patients were stratified into two groups based on pre-operative AET. Surgical selection criteria for patients prior to laparoscopic Nissen fundoplication included pre-operative testing with upper endoscopy, manometry, and ambulatory pH testing. All patients had a DeMeester score greater than 14.7 on ambulatory pH testing. Standardized surgical technique included high circumferential mediastinal mobilization of the esophagus, posterior cruroplasty with permanent interrupted suture, division of the short gastric vessels, and Nissen fundoplication around a 56 French dilator.

### Data collection

Using the electronic medical record, patient demographic information including age, sex, BMI, ASA, pre-operative GERD evaluation and operative details were recorded. Disease-specific quality of life measured with the GERD Health Related Quality of Life Scale (GERD-HRQL) was assessed pre-operatively, then at short-term (4–8 weeks), medium-term (6–36 months) and long-term (5 + years) follow-up. Additionally, patients were surveyed on their PPI use, overall satisfaction with the surgical outcome, and if they would have undergone the operation given current knowledge. All collected data was maintained in an institutionally reviewed and approved database.

### Outcome measures

The primary outcome of this study was long-term disease-specific quality of life as measured with the GERD-HRQL. The GERD-HRQL is a 10-question, Likert scale questionnaire that assesses heartburn severity, positional heartburn symptoms, heartburn after meals, changes in diet or sleep related to heartburn, dysphagia or odynophagia severity, bloating symptoms, and the burden of taking medication for reflux [15]. The maximum score is 50, with higher scores associated with worse burden of disease and poorer quality of life. The GERD-HRQL has been utilized as a disease-specific quality of life survey for 25 years in over 700 peer-reviewed studies [16]. Additional outcomes included PPI use, overall satisfaction with the surgical outcome, and if they would have, in hindsight, have undergone the operation. Satisfaction response options included “satisfied”, “neutral”, and “not satisfied”, and only “satisfied” responses were considered a positive response. Reoperation for recurrent reflux or complications related to surgery (such as dysphagia refractory to endoscopic intervention and reoperation for development of paraesophageal hernia) were also monitored. The time interval to follow-up was determined on the basis of the time at which that had an interval GERD-HRQL completed.

### Statistical analyses

Descriptive statistics and both univariate and multivariate logistic regression analyses were performed. On logistic regression, variables were modeled with the following parameters: age and BMI treated as continuous variables, sex coded as M or F, ASA coded as 1, 2, 3 or 4, and AET classification as borderline GERD or GERD. T-test was used to compare GERD-HRQL by AET cohort at each time point, and paired t-test utilized to compare GERD-HRQL scores between pre-operative baseline and each follow-up interval. *p* value < 0.05 was considered significant. All statistical analyses were performed using JMP 16 (*SAS Institute Inc, North Carolina, USA*).

## Results

225 patients meeting the study criteria underwent laparoscopic Nissen fundoplication for GERD during the study period. The mean age was 50.1 ± 13.4 years and 75% (*n* = 169) were female. 25 patients (11.1%) were stratified to the borderline GERD group (AET 4–6%) and 200 patients (88.9%) were stratified to the GERD group (AET ≥ 6%) (Table 1). Between cohorts, mean age, sex, BMI and ASA classification were not significantly different (*p* > 0.05). The mean DeMeester score and mean AET were significantly lower in the borderline GERD group than the GERD group.

**Table 1:** Baseline patient characteristics, by AET stratification

Patient demographics			
	Borderline GERD *N* = 25 (11.1%)	GERD *N* = 200 (88.9%)	*p*-value
Age	49 [39.5,63.5]	51 [41,58]	0.746
Female	76.0% (*n* = 19)	75.0% (*n* = 150)	0.932
BMI	29.5 [26.6,34.7] (kg/m^2^)	31.9 [28.4,36.1] (kg/m^2^)	0.123
ASA	2 [2, 3]	2 [2, 3]	0.627
DeMeester score	21.0 ± 5.1	52.7 ± 1.8	** < 0.001**
AET	5.2 ± 1.4	14.7 ± 0.5	** < 0.001**

Baseline patient characteristics of patient cohort who underwent Nissen fundoplication for GERD, stratified by borderline GERD (AET 4–6%) and GERD (AET ≥ 6%). Number indicates median [25% quartile, 75% quartile] or mean ± standard deviation for continuous variables. Statistically significant differences between groups are bolded

Disease-specific quality of life measured with the GERD Health Related Quality of Life Scale (GERD-HRQL) was assessed pre-operatively, then at short-term (5 weeks [IQR 4, 8]), medium-term (14 months [IQR 7.25, 31]) and long-term (81 months [IQR 66.5, 96]) follow-up (Table 2). The median follow-up duration at each interval was not statistically different between borderline GERD and GERD groups. The proportion of patients with post-operative GERD-HRQL survey adherence in each group at short-term follow-up was 84% vs 94%, at medium-term follow-up was 68% vs 76.5%, and at long-term follow-up was 48% vs 45.5%.

**Table 2:** Postoperative follow-up data

	Borderline GERD	GERD	*p*-value
Short-term			
Respondents (*n*, %)	21, 84%	188, 94%	
Weeks to follow-up (median [IQR])	5 [4,8]	5 [4, 8]	0.550
Medium-term			
Respondents (*n*, %)	17, 68%	153, 76.5%	
Months to follow-up (median [IQR])	20 [7,36]	13 [7.5,30.5]	0.509
Long-term			
Respondents (*n*, %)	12, 48%	91, 45.5%	
Months to follow-up (median [IQR])	83.5 [67.3,99]	79 [66.5,94.5]	0.455

Number of patients with interval GERD-HRQL follow-up and the median interval follow-up duration, by weeks or months, stratified by borderline GERD (AET 4–6%) and GERD (AET ≥ 6%). Number indicates median [25% quartile, 75% quartile] for continuous variables. Statistically significant differences between groups are bolded

Baseline GERD-HRQL was lower in the borderline GERD group (24.3 vs 30.0, *p* = 0.031) (Table 3). Borderline GERD was weakly associated with short-term PPI use (*R*^2^ = 0.053, *p* = 0.028). GERD-HRQL scores did not differ between borderline GERD and GERD patients at short-(6.0 vs 7.1, *p* = 0.630), medium-(12.0 vs 12.1, *p* = 0.818), or long-term follow-up (10.0 vs 9.0, *p* = 0.757). At long-term follow-up there was no difference between groups in PPI use (50% vs 47%, *p* = 0.852), satisfaction (58% vs 76%, *p* = 0.187), willingness to repeat the procedure given the benefit of hindsight (75% vs 85%, *p* = 0.386), or need for reoperation (14% vs 13%, *p* = 0.910), as both univariate and multivariate analysis adjusted for age, sex, ASA, and BMI (*p* > 0.05) (Table 4). The absolute change in GERD-HRQL from pre-operative baseline was statistically significant for both groups at all follow-up intervals except for medium-term follow-up in the borderline GERD group. Comparing the absolute change in GERD-HRQL from before to after surgery, at long-term follow-up the borderline GERD group experienced at 12 point improvement, whereas the GERD group experienced at 21 point improvement.

**Table 3:** Long-term operative outcomes

	Borderline GERD	GERD	*p*-value
Pre-operative			
GERD-HRQL	24.3 ± 2.5	30.0 ± 0.8	**0.031**
Short-term			
GERD-HRQL	6.0 ± 2.1	7.1 ± 0.7	0.630
Δ GERD-HRQL (p-value)	−19.6 (**< 0.001)**	−22.6 (**< 0.001)**	
PPI use	18.2%	4.4%	**0.028**
Medium-term			
GERD-HRQL	12.0 ± 4.3	12.1 ± 1.5	0.818
Δ GERD-HRQL (p-value)	−14.7 (0.068)	−19.2 (**< 0.001)**	
PPI use	18.2%	22.2%	0.760
Satisfaction	85.7%	71.0%	0.408
Hindsight	80.0%	87.8%	0.625
Long-term			
GERD-HRQL	10.0 ± 2.9	9.0 ± 1.1	
Δ GERD-HRQL (p-value)	−12.3 (**0.022)**	−21.3 (**< 0.001)**	0.757
PPI use	50.0%	47.1%	0.852
Satisfaction	58.3%	76.1%	0.187

Operative outcomes of patient cohort who underwent Nissen fundoplication for GERD, stratified by borderline GERD (AET 4–6%) and GERD (AET ≥ 6%) and time of follow-up. Delta GERD-HRQL as paired t-test when compared to pre-operative baseline. Statistically significant differences between groups are bolded

**Table 4:** Long-term adjusted outcomes for patients with borderline GERD compared to GERD

	Odds ratio (95% CI)	*p*-value
PPI use	0.93 (0.27–3.22)	0.910
Satisfaction	1.94 (0.53–7.15)	0.317
Hindsight	1.52 (0.34–6.79)	0.583
Reoperation	1.03 (0.19–5.51)	0.974

Odds ratio and 95% confidence interval of active PPI use, satisfaction with operation, willingness to undergo surgery with benefit of hindsight, and need for reoperation at long-term follow-up for patients with borderline GERD compared to GERD, on adjusted analysis for age, sex, ASA and BMI

**Fig. 1:**
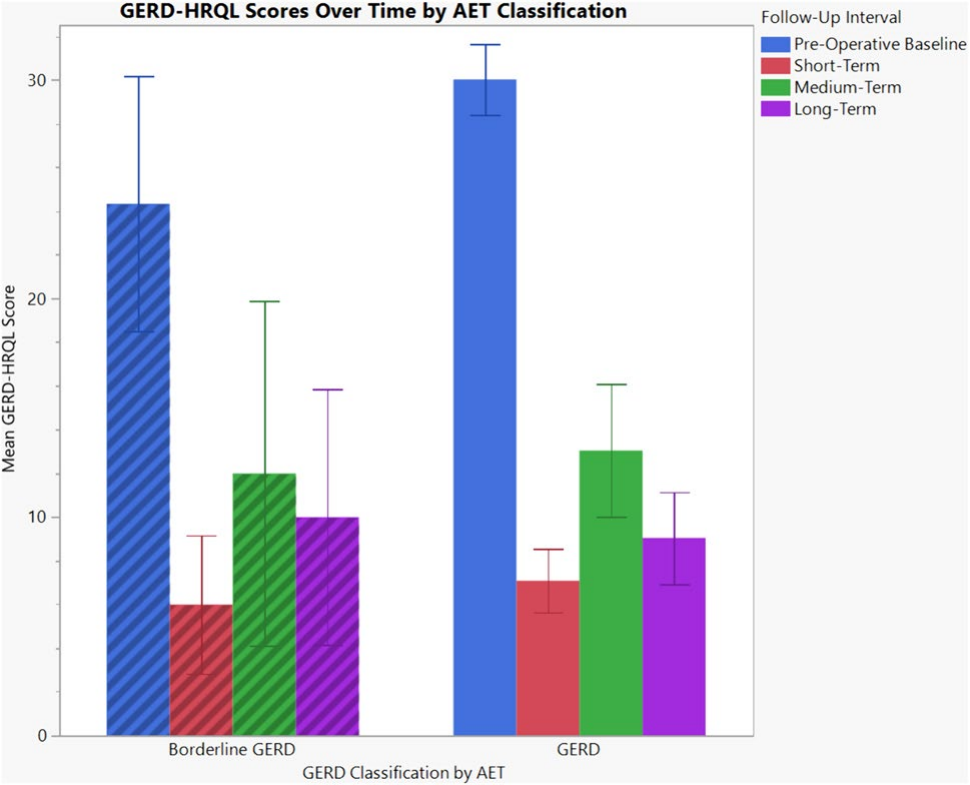
GERD-HRQL scores over time after Nissen fundoplication, by pre-operative AET classification. Error bars represent 95% confidence interval of the mean

Comparing patients with borderline GERD and GERD, as stratified by pre-operative AET, both groups demonstrated sustained and statistically significant improvements in GERD-HRQL after surgery to a long-term follow-up of seven years (Fig. 1).

## Discussion

There is a paucity of data regarding long-term quality of life outcomes after laparoscopic Nissen fundoplication for patients with borderline GERD as defined by AET. Our study demonstrated that patients with borderline GERD and GERD report significant GERD-HRQL improvements at 7 years following laparoscopic Nissen fundoplication. They also demonstrate similar long-term PPI usage, satisfaction with surgical results, willingness to repeat the surgery given the benefit of hindsight, and rates of reoperation.

Our findings are congruent with other reports of QOL improvements after fundoplication. Multiple prior retrospective studies of disease-specific quality of life scores after laparoscopic fundoplication (including Dor, Toupet and Nissen) demonstrate significant and sustained improvements to follow-up intervals of up to 10 years [8, 9, 11]. Other studies assessing patient satisfaction and willingness to undergo operation given benefit of hindsight have found rates between 83 and 90% at 20 years follow-up, which are comparable to our hindsight rate of 75–85%, but higher than our patient satisfaction rate of 58% in the borderline GERD group [10, 11, 13]. Notably most of these ultra-long-term follow-up studies were performed in Europe, and differences in expectations and patient experience may impact subjectively measured satisfaction rates.

It is interesting to note that while the absolute scores on GERD-HRQL for both borderline and GERD cohorts are equivalent at each follow-up interval, the pre-operative baseline score is significantly lower for the borderline GERD group. On average, patients with borderline GERD have less severe symptoms, which is not an unexpected finding. As such, the absolute improvement in symptoms for the borderline GERD group is smaller. Patients with borderline GERD who are being counseled on potential operative anti-reflux intervention should be told that they are likely to benefit from surgery, but potentially less so than patients with more severe symptoms. Prior studies of GERD-HRQL improvements after fundoplication have noted absolute score improvements of over 50% the original score, with an aggregate of 150 studies demonstrating an average improvement of 18 points after surgical anti-reflux procedure [15, 16]. For our cohort, though all patients had significant improvements in their GERD-HRQL, the absolute improvement after surgery at long-term follow-up was 12 points for the borderline group compared to 21 points for the GERD group. While counseling patients who meet the definition of GERD by DeMeester score but are borderline by AET, it is important to emphasize that the absolute improvement in their symptoms is likely to be less than that of someone with higher baseline disease severity, simply because there is less room for improvement.

A retrospective study by Amundson et al. in 2023 assessing a similar question of whether or not patients with borderline GERD benefit from anti-reflux surgery concluded that AET may be a better criterion than DeMeester score as a component of operative candidacy [14]. They followed a patient cohort who had undergone a mix of fundoplication (Nissen, Toupet, or Dor), magnetic sphincter augmentation, and endoscopic anti-reflux mucosectomy. For the patients who underwent fundoplication, they found that patients who did not meet new AGA criteria for GERD had worse atypical reflux symptoms on follow-up, and worse GERD-HRQL scores that did not reach statistical significance. When compared to our results, which were specific to Nissen fundoplication, we found that at all levels of follow-up, including at medium-term (median 14 months) and long-term (median 6.8 years) intervals, the GERD-HRQL scores were equivalent between borderline and GERD groups. In addition, the number of patients in our fundoplication cohort with the longest follow-up totaled 103, which may have allowed sufficient data to achieve statistical significance when compared to 32 fundoplication patients with 2-year follow-up in the other study. Our study builds on the body of knowledge regarding long-term quality of life outcomes in borderline GERD patients. As surgical therapy for GERD is frequently performed for quality of life reasons, the critical outcome to determine success and impact of surgery is long-term improvements in quality of life.

Our data supplements previous studies by controlling for operative technique with a single-surgeon, single-institution database of laparoscopic Nissen fundoplication, and with a long follow-up duration. We were also able to capture quality of life scores at several intervals to observe trends over time. Further investigation into the long-term outcomes of both operative and endoscopic anti-reflux procedures in patients with borderline GERD will add to our understanding of which patient cohorts benefit from intervention. Investigation into different pre-operative GERD phenotypes and improvements in typical versus atypical GERD symptoms in patients with borderline objective testing remain understudied.

There is ongoing discussion within the medical community regarding the best objective measurements to diagnose GERD. Though the 2022 AGA guidelines recommend the diagnosis of GERD based on AET [4], there is an argument in support of classifying patients with borderline GERD by AET as having GERD if DeMeester score is positive [17]. The consensus definition of GERD stratifies patients into candidacy for surgical and endoscopic anti-reflux treatment, and improving the understanding of which patient cohorts experience long-term benefits is critical for setting guidelines on who should be offered surgery. This study investigates patients who meet GERD criteria by DeMeester score but do not by AET criteria, which does not apply broadly to all patients who are classified as borderline GERD by AET. As the AET criteria are more broadly adopted in the medical community and referral patterns for surgical correction of GERD change, we anticipate increasing opportunities to directly compare outcomes for patients with borderline GERD who do or do not undergo surgical correction.

### Limitations

This study is limited by retrospective design and the fact that all patients in the study cohort were treated in a single institution, which limits the generalizability of the study results. Our satisfaction and reoperation rates are similar when compared to existing studies of outcomes after laparoscopic fundoplication, suggesting validity beyond our patient population. The exclusion of re-operative fundoplication was due to the goal of limiting confounding factors; this intentional study design limits the utility of these results of patients requiring re-do anti-reflux surgery. Similarly, the decision to include only laparoscopic Nissen fundoplication was to control for potential confounding related to operative approach but limits the generalizability of the study results to other anti-reflux procedures. Our institution does not routinely perform post-operative objective acid testing after anti-reflux surgery, so we do not have post-operative AET or DeMeester scores except in patients who reported recurrence of GERD symptoms, though there is a subset of patients who re-initiate PPIs who may benefit from further objective investigation.

## Conclusion

Fundoplication for GERD has been well-established to provide durable improvements in disease-specific quality of life for appropriately selected patients. The new AGA guidelines for diagnosis of GERD versus borderline GERD provide an alternative stratification strategy that could adversely impact a patient’s access to quality of life improving surgical fundoplication. While individualized treatment approaches must consider all available subjective and objective testing data, particularly prior to surgical therapy, AET must be evaluated in the context of other objective testing data and symptoms. Although the magnitude of symptomatic improvement may be less than those with an AET > 6%, patients with borderline GERD (AET 4–6%) exhibit significant long-term patient-reported quality of life outcome improvements after laparoscopic Nissen fundoplication.
